# Finite element analysis of the femoral diaphysis of fresh-frozen cadavers with computed tomography and mechanical testing

**DOI:** 10.1186/s13018-018-0898-7

**Published:** 2018-07-31

**Authors:** Yasushi Wako, Junichi Nakamura, Yusuke Matsuura, Takane Suzuki, Shigeo Hagiwara, Michiaki Miura, Yuya Kawarai, Masahiko Sugano, Kento Nawata, Kensuke Yoshino, Sumihisa Orita, Kazuhide Inage, Seiji Ohtori

**Affiliations:** 1 Department of Orthopedic Surgery, Graduate School of Medicine, Chiba University, 1-8-1 Inohana, Chuo-ku, Chiba City, Chiba 260-8677 Japan; 2 Department of Bioenvironmental Medicine, Graduate School of Medicine, Chiba University, 1-8-1 Inohana, Chuo-ku, Chiba City, Chiba 260-8677 Japan

**Keywords:** Finite element analysis, The femoral diaphysis fracture, Validation study, Fresh frozen cadaver, Computed tomography

## Abstract

**Background:**

The purpose of this study was to validate a diaphyseal femoral fracture model using a finite element analysis (FEA) with mechanical testing in fresh-frozen cadavers.

**Methods:**

We used 18 intact femora (9 right and 9 left) from 9 fresh-frozen cadavers. Specimens were obtained from 5 males and 4 females with a mean age of 85.6 years. We compared a computed tomography (CT)-based FEA model to diaphyseal femoral fracture loads and stiffness obtained by three-point bending. Four material characteristic conversion equations (the Keyak, Carter, and Keller equations plus Keller’s equation for the vertebra) with different shell thicknesses (0.3, 0.4, and 0.5 mm) were compared with the mechanical testing.

**Results:**

The average fracture load was 4582.8 N and the mean stiffness was 942.0 N/mm from actual mechanical testing. FEA prediction using Keller’s equation for the vertebra with a 0.4-mm shell thickness showed the best correlations with the fracture load (*R*^2^ = 0.76) and stiffness (*R*^2^ = 0.54). Shell thicknesses of 0.3 and 0.5 mm in Keller’s equation for the vertebra also showed a strong correlation with fracture load (*R*^2^ = 0.66 for both) and stiffness (*R*^2^ = 0.50 and 0.52, respectively). There were no significant correlations with the other equations.

**Conclusion:**

We validated femoral diaphyseal fracture loads and stiffness using an FEA in a cadaveric study.

## Background

Although proximal femoral fractures are common in the elderly, femoral shaft fractures are seen in all generations. Because of the difference of fracture type and bone quality, we may need individual consideration of proximal femoral fracture and femoral diaphyseal fracture.

Recently, computed tomography-based finite element analysis (CT-based FEA) has been widely used for mechanical analysis of the femur. Many reports have described fracture models of the femur following traffic accidents and simulations of stress distribution after joint replacement [1–13]. When we consider a femoral diaphyseal fracture and the stress distribution for femoral diaphyseal after prosthesis replacement using FEA, the whole femoral shaft must be evaluated. However, there are no reports of FEA models of diaphyseal femoral fractures. Although experimental data can be used to validate CT-based FEA models, most of the fracture models in previous reports were of the proximal femur [1–6]. The proximal of femur have abundant cancellous bone, while the femoral diaphysis mainly consists of cortical bone. The bone architecture of the proximal femur and the diaphysis are different, and thus, they should be examined separately.

The aim of this study is to validate the newly constructed CT-based FEA models of the femur by comparing with the data obtained from the actual mechanical fracture tests using the original fresh-frozen cadaveric femurs.

## Methods

The research protocol was in compliance with the Helsinki Declaration; it was approved by the Research Ethics Committee of our institution and registered with the University Hospital Medical Information Network. Written informed consent was obtained from all the donors before death.

### Specimens

**Fig. 1:**
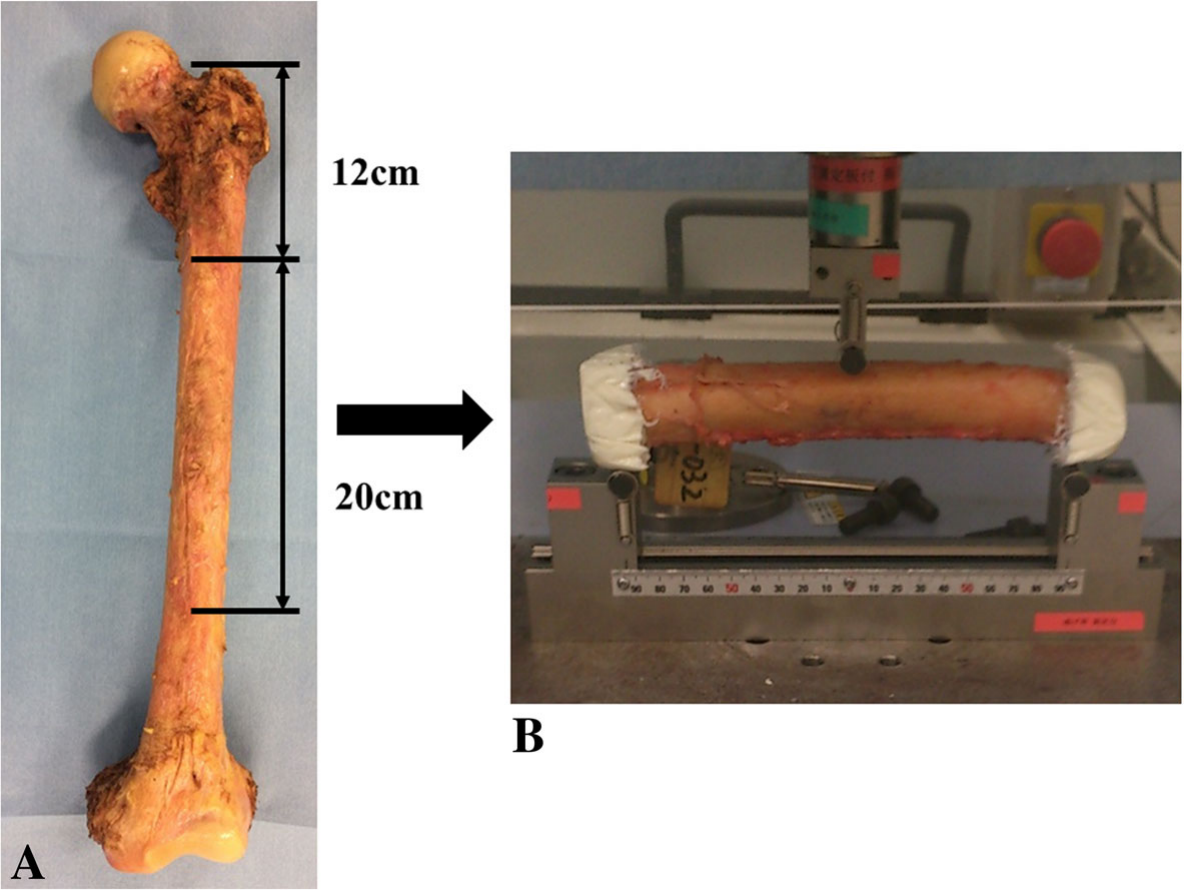
Process of mechanical testing. **a** Whole femurs retrieved from the cadaver were sawed 12 cm distal to the tip of the greater trochanter and 20 cm distal to the first cut. **b** Both ends of the femoral diaphysis were fixed using resin cement. A compressive force was applied by three-point bending

In the present study, 18 intact femurs from 9 fresh-frozen cadavers (5 men and 4 women) were used. Cadavers were provided from the Clinical Anatomy Laboratory of our institution. The mean age at death was 85.6 years (range, 74–98 years). All of the cadavers were kept at − 22 °C and were thawed at room temperature immediately before the tests. They were not refrozen. After retrieval of the femur, all soft tissues were removed. Prior to the tests, the femurs were imaged using CT (Aquilion ONE, Toshiba Medical Systems, Tokyo, Japan; 320-row detector, 120 kV, 200 mA, pixel width 0.3 mm, slice thickness 0.5 mm) with a phantom for calibration (QRM-BDC, QRM, Möhrendorf, DE) that contained three rods made of hydroxyapatite (0, 100, and 200 mg/cm^3^). Then, to obtain specimens of the femoral diaphysis, the femurs were sawed 12 cm distal to the proximal tip of the greater trochanter and 20 cm distal to the first cut (Fig. 1a). The specimens were kept moist during the procedure.

#### Mechanical tests

The specimens were loaded using the Autograph AG-20000N X Plus Precision Universal Tensile Tester (Shimadzu, Kyoto, Japan). The proximal and the distal 2.5-cm portions of the specimen were embedded in resin cement boxes shaped square parallel to the cut surface. A three-point bending test was done without fixing the resin cement boxes to the jig (Fig. 1b). A compression load was applied at the midpoint of the specimen on the anterior surface of the femoral shaft with a crosshead speed of 5 mm/min. Load was applied until the yield point was confirmed on the load-deformation curve, which was continuously recorded in 100 Hz throughout the mechanical tests. Fracture was defined by a rapid decrease on the load-displacement curve. The yield point was considered as the time point where a fracture of the specimen occurred. The load recorded at the yield point was defined as the fracture load. The stiffness was calculated as the slope of the load-deformation curve between 20 and 80% of the fracture load reference to the previous study that was reported by Miura et al. [1].

### Non-linear FEA study

#### Model development

CT-based morphological data were transmitted to an HP Z400 workstation (Hewlett-Packard, Palo Alto, CA, USA). A non-linear FEA model of the femur was constructed from the CT data using Mechanical Finder software (Research Center for Computational Mechanics, Tokyo, Japan). The FEA model was made to exactly match the specimen used for the mechanical test (proximal end, 12 cm distal to the tip of the greater trochanter; distal end, 20 cm distal to the proximal end). The cancellous bone and the inner cortical bone were simulated with 3-mm linear tetrahedral elements according to the previous reports [5], and the outer cortical bone was constructed with 3 × 3 × 0.3–0.5 mm triangular shell elements. The shell elements were to reinforce the margin of the bone, because the tetrahedral elements of the bone margin might show lower HU values as they calculated the average of HU values in the element. Three models were created for each specimen with different shell thicknesses of 0.3, 0.4, and 0.5 mm.

#### Material properties

To allow for bone heterogeneity, the material properties of each element were calculated using the Hounsfield unit (HU) value at their location. The ash density of each element was set as the mean ash density of the voxels contained within the corresponding element. Young’s modulus and yield stress of each tetrahedral element, assumed to be isotropic, were calculated from the equations proposed by Keyak [2, 3, 14], Carter [15], Keller, and Keller for vertebra [16] (Table 1). Moduli lower than 0.01 MPa were assigned a new value of 0.01 MPa, and those higher than 20 GPa got a new value of 20 GPa, and Young’s modulus and yield stress of the shell element were calculated using a CT value of 1500 HU reference to the previous study that was reported by Miura et al. and Matsuura [1, 17]. The Drucker-Prager equivalent criterion was adopted for the yield of the element. [18]. The Poisson’s ratio for each element was set at 0.3 according to the previous reports [19].

**Table 1:** Equations proposed by Keyak, Carter, Keller, and Keller for vertebra

	Young’s modulus (*E*: MPa)	Yield stress (*σ*: MPa)
Keyak	*E* = 0.001 (*ρ* = 0)	*σ* = 1.0 × 10^20^ (*ρ* ≦ 0.2)
	*E* = 33,900 *ρ*^2.20^ (0 < *ρ* ≦ 0.27) *E* = 5307 *ρ* + 469 (0.27 < *ρ* < 0.6) *E* = 10,200 *ρ*^2.01^ (0.6 ≦ ρ)	*σ* = 137 *ρ*^1.88^ (0.2 < *ρ* < 0.317), *σ* = 114*ρ*^1.72^ (0.317 ≦ *ρ*)
Carter	*E* = 0.001 (*ρ* = 0)	*σ* = 1.0 × 10^20^ (*ρ* ≦ 0.2)
	*E* = 3790(0.01)^0.05^ *ρ* (*ρ* < 0)	*σ* = 68(0.01)^0.06^ *ρ*^2^ (0.2 < *ρ*)
Keller	*E* = 0.001 (*ρ* = 0)	*σ* = 1.0 × 10^20^ (*ρ* ≦ 0.2)
	*E* = 10,500 *ρ*^2.51^ (*ρ* < 0)	*σ* = 117 *ρ*^1.93^ (0.2 < *ρ*)
Keller for vertebra	*E* = 0.001 (*ρ* = 0)	*σ* = 1.0 × 1020 (*ρ* ≦ 0.2)
	*E* = 1890 *ρ*^1.92^ (*ρ* < 0)	*σ* = 284 *ρ*^2.27^ (0.2 < *ρ*)

*E* Young’s modulus (MPa), *σ* yield stress (MPa), *ρ* ash density (g/cm^3^)

#### Others

**Fig. 2:**
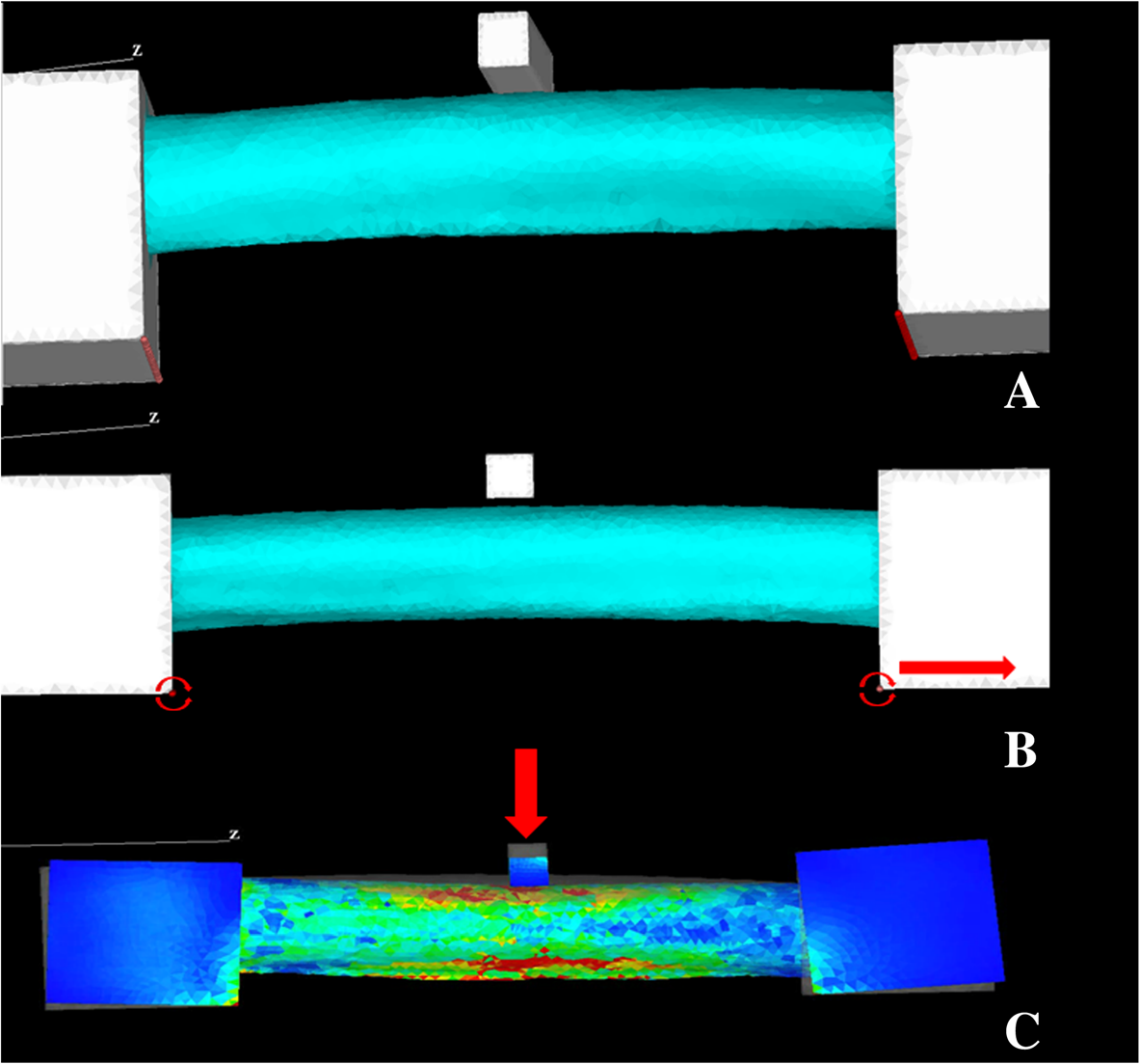
Process of finite element analysis (FEA). **a** The resin boxes were restrained from their bottom edges to their centers. **b** Rotations of both edges around the restraint axis were allowed. One of the edges was allowed to move only in the bone-axis direction to reproduce the motion of the bone in bending. **c** A compressive load was applied through the stainless steel bar at the center of the beam on the anterior aspect of the femoral diaphysis

The proximal and the distal ends of the FEA model were completely fixed with 2.5-cm-wide resin boxes. The resin boxes were restrained along the medial bottom edge, but the edge of one resin box was allowed to move only in the bone axis direction. Rotation around both edges was allowed (Fig. 2a, b). A compressive force was applied through a stainless steel bar at the center of the model on the anterior surface of the femoral shaft (Fig. 2a). Interface conditions between the bone and resin were set as the bonded condition, and those between the bone and stainless steel as the contact condition. The coefficient of friction was set at 0 at the contact point. The reaction force and the amount of displacement on a stainless steel bar were recorded at each point. Based on the FEA results, a load-deformation curve was constructed, and the fracture load was identified by a rapid decrease in load. The FEA-predicted stiffness was defined the same as for the mechanical test (a slope between 20 and 80% of the fracture load).

#### Statistical analysis

FEA results were compared with the results of mechanical tests using Pearson’s correlation coefficients. Influence of the shell thickness was evaluated for the most highly correlated Keller-vertebra equation. A *p* value of less than 0.05 was considered statistically significant (BellCurve for Excel, Social Survey Research Information Co., Ltd. Tokyo, Japan).

## Results

All femoral fractures occurring in mechanical tests were transverse fractures at the center of the femoral shafts. The FEA model also reproduced the same fracture site.

### Fracture load

**Fig. 3:**
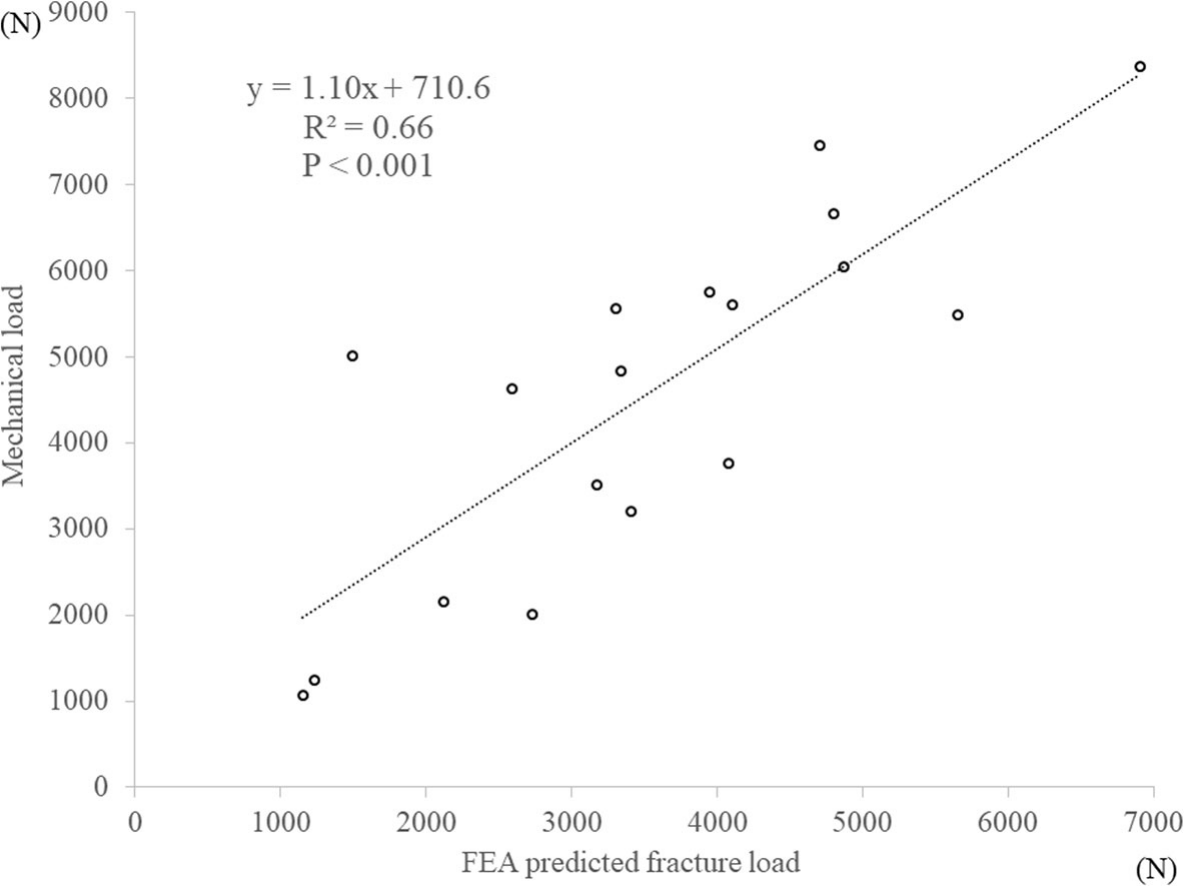
Correlation of fracture load between actual mechanical testing and the FEA prediction using the Keller-vertebra equation with a 0.3-mm shell thickness, (mechanical load) = 1.097 × (FEA-predicted fracture load) + 710.6, *R*^2^ = 0.66, *p* < 0.001

The average fracture load was 4582.8 N (SD 2019.4) in the mechanical tests. Using a 0.3-mm outer cortex, the data were not correlated to the estimated value from the three FEA equations by Keyak, Carter, or Keller (*R*^2^ = 0.013, 0.056, 0.039, *p* = 0.66, 0.35, 0.44, respectively). On the other hand, there was a significant linear correlation with the predicted load with Keller’s equation for the vertebra (Keller-vertebra equation) (*R*^2^ = 0.66, *p* < 0.001, Fig. 3).

### Stiffness

**Fig. 4:**
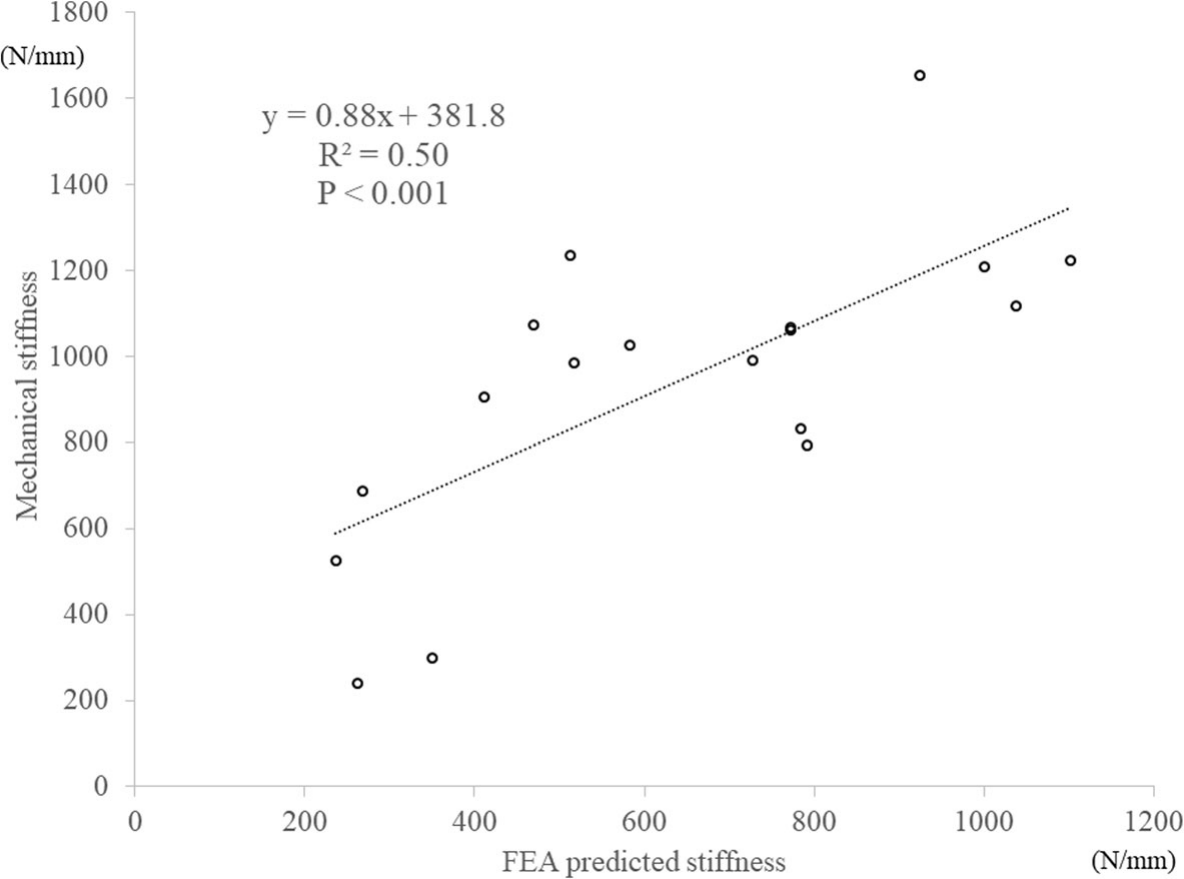
Correlation of stiffness between actual mechanical testing and the FEA prediction using the Keller-vertebra equation with a 0.3-mm shell thickness, (mechanical stiffness) = 0.876 × (FEA-predicted stiffness) + 381.8, *R*^2^ = 0.52, *p* < 0.001

The average stiffness was 942.0 N/mm (SD 335.0) in the mechanical tests. There were no significant correlations with the FEA prediction using a 0.3-mm shell in Keyak’s, Carter’s, or Keller’s equations (*R*^2^ = 0.013, 0.17, 0.031, respectively). However, there was a strong linear correlation with the value estimated with the Keller-vertebra equation (*R*^2^ = 0.50, *p* < 0.001, Fig. 4).

### Shell thickness effect

**Fig. 5:**
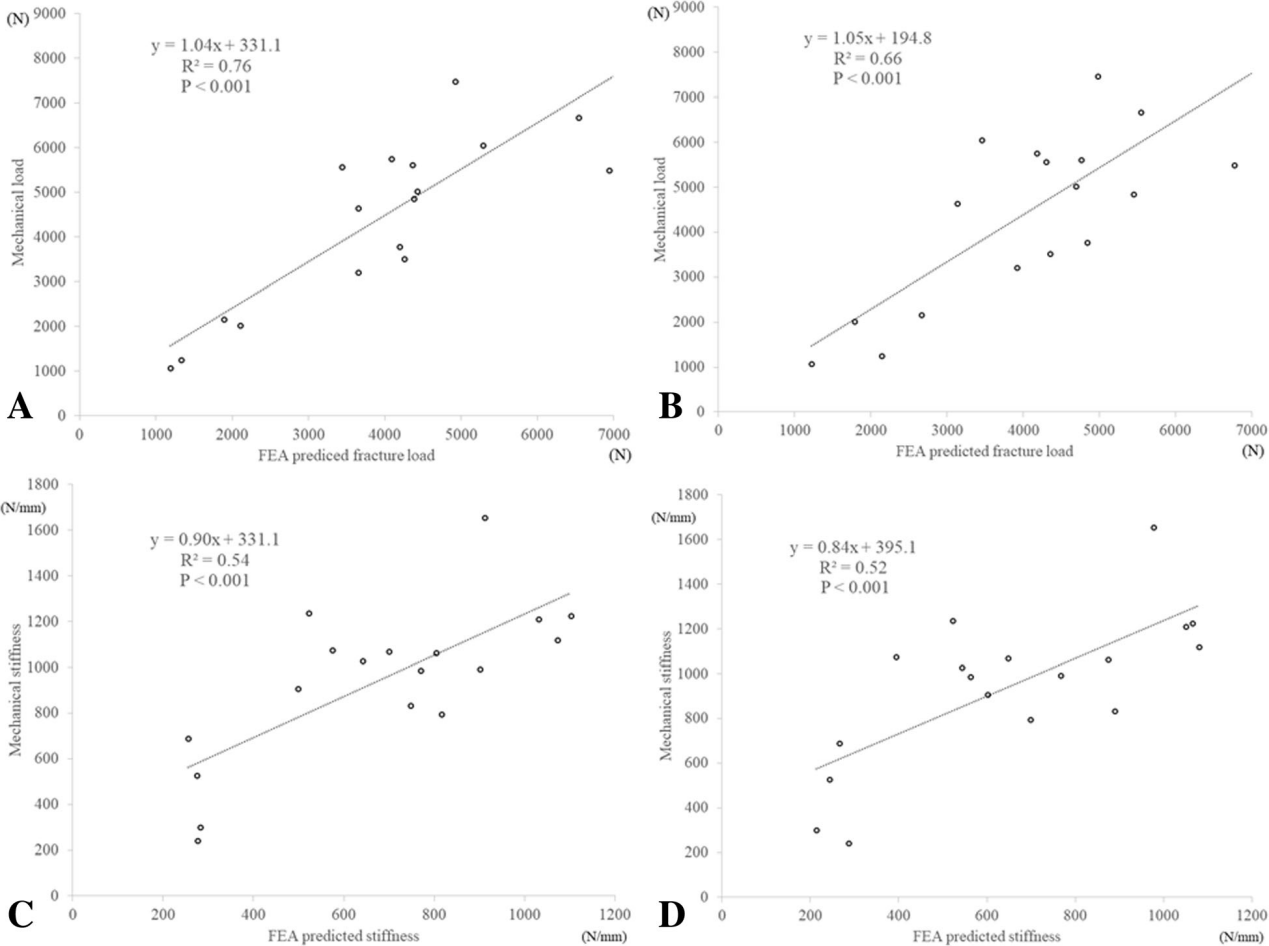
Shell thickness effect on fracture loads and stiffness using the Keller-vertebra equation. **a** Fracture load with a 0.4-mm shell thickness. (Mechanical load) = 1.037 × (FEA-predicted fracture load) + 331.1, *R*^2^ = 0.76, *p* < 0.001. **b** Fracture load with a 0.5-mm shell thickness. (Mechanical load) = 1.047 × (FEA-predicted fracture load) + 194.8, *R*^2^ = 0.66, *p* < 0.001. **c** Stiffness with a 0.4-mm shell thickness. (Mechanical stiffness) = 0.903 × (FEA-predicted stiffness) + 331.1, *R*^2^ = 0.54, *p* < 0.001. **d** Stiffness with a 0.5-mm shell thickness. (Mechanical stiffness) = 0.843 × (FEA-predicted stiffness) + 395.1, *R*^2^ = 0.52, *p* < 0.001

The average fracture loads with 0.4- and 0.5-mm shell thickness models predicted by the Keller-vertebra equation were 4100.9 N (SD 1694.8) and 4202.4 N (SD 1545.6), respectively. Both showed a good linear correlation with the results of the mechanical tests (*R*^2^ = 0.76 and 0.66, both *p* < 0.001, respectively, Fig. 5a, b). The mean stiffness with 0.4 and 0.5-mm shell thickness models estimated by the Keller-vertebra equation was 676.9 N/mm (SD 273.0) and 649 N/mm (SD 287.0), respectively. Both showed good linear correlations with the results of the mechanical tests (*R*^2^ = 0.54 and 0.52, both *p* < 0.001, respectively, Fig. 5c, d). A shell thickness of 0.4 mm correlated better than the 0.3- or 0.5-mm thicknesses for both fracture load and stiffness.

## Discussion

The FEA was developed in 1970, and three-dimensional analysis using CT started around 1990 [20]. In recent years, the FEA has been widely used in the field of orthopedic surgery. There are several validation studies reported, including those for the proximal femur, the vertebra, and the distal radius [1–3, 5, 17, 21–23]. In validation studies of the proximal femur, Bessho et al. and Keyak reported a strong positive correlation between the FEA results and actual mechanical tests (*R*^2^ = 0.96 and 0.94, respectively) [2, 5]. For the vertebra and distal radius, Imai et al. and Matsuura et al. also reported positive correlations (*R*^2^ = 0.96 and 0.97, respectively) [17, 21]. However, to our knowledge, a validation study for the femoral diaphysis has not been reported. In the present study, the FEA showed significant correlations with the femoral shaft fracture load (*R*^2^ = 0.76) and stiffness (*R*^2^ = 0.54) calculated using the Keller-vertebra equation with a 0.4-mm shell thickness. The correlation coefficients in our study were not as high as those in the previous reports [2, 5, 17, 21]. It may be due to the fact that those studies excluded an analysis of the stiffness. In the studies that investigated stiffness, the values were similar to the current study. Dall'Ara et al. [6] showed strong similarity between the FEA and actual mechanical tests for fracture load (*R*^2^ = 0.72) and stiffness (*R*^2^ = 0.54) in a validation study of the proximal femur. In a vertebral study, Matsuura et al. also reported lower values for fracture load (*R*^2^ = 0.48) and stiffness (*R*^2^ = 0.79). We believe this is the first report on a successful validation of the FEA model of the femoral diaphysis.

Keyak’s equation has been widely used in the previous FEA reports [2, 3, 5, 9–11, 21]. However, we could not find any correlation between the mechanical tests and values produced by the Keyak equation, nor by Carter’s or Keller’s equations, except for the Keller-vertebra equation. To explain this, we calculated stiffness and fracture load using two equations, Keyak’s equation and the Keller-vertebra equation. The ash density of cortical bone of the femoral diaphysis was substituted with 0.935 g/cm^3^ by reference to Bousson’s report [24]. As a result, stiffness showed about a fivefold difference between the Keyak and the Keller-vertebra equations (8911 and 1661 MPa, respectively). Moreover, the fracture load calculated using Keyak’s equation was less than half of that using Keller’s equation for the vertebra (101 and 243 MPa, respectively). Therefore, Keyak’s bone model seems too stiff to reproduce elasticity in bending. Using Keyak’s model, the fractures likely occur at an early stage due to the low yield stress. Although many studies have used Keyak’s equation, the present study suggests that it may provide an inaccurate estimate of the mechanical properties of the femoral diaphysis. We propose that the Keller-vertebra equation is a suitable material characteristic conversion equation for the FEA of the femoral diaphysis when an FEA of femoral diaphyseal fractures is applied to examine stress distribution.

The shell thickness has often been defined as 0.3–0.4 mm in previous validation studies [5, 17, 23]. However, the optimum shell thickness is unknown. The current study showed that a shell thickness of 0.4 mm had the best correlation coefficient for the femoral diaphysis both for fracture load and stiffness. Further study is needed to determine the optimum shell thickness for other bone sites.

The present study has several limitations. First, only fractures from three-point bending tests were evaluated. Because of the limited number of cadavers, we could not perform other fracture tests. Clinically, there are various types of femoral diaphyseal fractures. Further study is necessary to validate our FEA for other types of fractures, such as spiral fractures or those from axial compression forces. Secondly, the steel bar model might not be made to exactly match the bar used for the mechanical test. But, in both cases, the load was applied to the center point of the femoral diaphyseal, and the behavior of the bone was similar, so we thought that the research had no influence. Third, all cadavers were from elderly individuals. It is possible that the fracture load or stiffness would be different in younger individuals. However, it is difficult to obtain femurs from young people. We studied matched pairs of right and left femurs in nearly equal numbers of men and women, and we observed high reproducibility without differences of laterality (data not shown). Thus, we think that the FEA of the femoral diaphysis is predictive of mechanical properties regardless of laterality or gender.

## Conclusion

In conclusion, we have confirmed that FEA can predict fracture loads of the femoral diaphysis. We propose that Keller’s equation for the vertebra with a 0.4-mm shell thickness allows prediction of fracture load and stiffness.
